# Nivolumab dose selection: challenges, opportunities and lessons learned for cancer immunotherapy

**DOI:** 10.1186/2051-1426-3-S2-P141

**Published:** 2015-11-04

**Authors:** Shruti Agrawal, Yan Feng, Amit Roy, Georgia Kollia, Brian Lestini

**Affiliations:** 1 Bristol-Myers Squibb, Princeton, NJ, United States

## Background

Dose selection for immuno-oncology (I-O) treatments presents unique challenges and opportunities. Surrogate efficacy endpoints in early clinical studies (typically objective anti-tumor response) may not fully represent the potential for improved overall survival in pivotal trials, complicating selection of a Phase III dose and schedule. Conversely, the immune-stimulating mechanism of action of I-O agents, such as nivolumab, a programmed death-1 (PD-1) immune checkpoint inhibitor, may support one dosing regimen across multiple tumor types. Here, we describe an integrated approach to identify the most suitable monotherapy dose for nivolumab across solid tumor indications.

## Methods

The dose was selected primarily based on nivolumab anti-tumor activity and safety data from a large Phase Ib open-label, dose-escalation study of nivolumab in 306 patients (pts) with advanced or recurrent malignancies including a total of 270 pts with melanoma (MEL), non-small cell lung cancer (NSCLC), or renal cell carcinoma (RCC) (CA209003) treated at doses from 0.1 to 10 mg/kg every two weeks (Q2W). In total, 306 pts were evaluable for safety across 5 tumor types, and 270 for efficacy in the select tumor types. Additionally, an integrated quantitative analysis was performed to estimate the relationships of dose-exposure response to biomarkers (receptor occupancy [RO]), safety (adverse events [AE] leading to discontinuation), and efficacy (objective response rate [ORR], progression free survival [PFS], and tumor growth dynamics).

## Results

Nivolumab was well tolerated up to the highest dose of 10 mg/kg administered in the dose escalation study, without identifying a maximum tolerated dose. The nature, frequency, and severity of any causality and treatment-related safety events were similar across tumor types and dose levels, as were probabilities of AEs leading to discontinuation. ORRs were similar between 1 and 10 mg/kg in MEL and RCC, however higher ORRs were observed in NSCLC at 3 and 10 mg/kg doses versus 1 mg/kg (Table 1). Peripheral RO was saturated at ≥0.3 mg/kg dose. In exposure-response analysis, a trend was observed for each tumor type but appeared to plateau at ≥3 mg/kg Q2W. While the exposure-response for tumor shrinkage was relatively flat, tumor progression rate tended to decrease with increasing exposure.

**Table 1:** 

	MEL (N=107)		NSCLC (N=129)		RCC (N-34)	
Dose (mg/kg)	ORR % (n/N)	PFS 24 wks, %	ORR % (n/N)	PFS 24 wks, %	ORR % (n/N)	PFS 24 wks, %

0.1	35 (6/17)	41	-	-	-	-
0.3	28 (5/18)	35	-	-	-	-
1	31 (11/35)	51	3 (1/33)	26	28 (5/18)	50
3	41 (7/17)	55	24 (9/37)	40	-	-
10	20 (4/20)	35	20 (12/59)	33	31 (5/16)	67

## Conclusions

The selected monotherapy dose of 3 mg/kg Q2W provides for unified dosing of nivolumab across the clinical development program. The approach taken, integrating dose-exposure responses with safety and efficacy data, represents a means to make rational dose selections for I-O agents.

